# Pre-hospital care after return of spontaneous circulation: Are we achieving our targets?

**DOI:** 10.1016/j.resplu.2024.100691

**Published:** 2024-06-21

**Authors:** I.A. Vos, F.G. Lucassen, B.W.J. Bens, B. Dercksen, R. Postma, E.M.F. Jorna, J.C. ter Maaten, M.M.R.F. Struys, E. ter Avest

**Affiliations:** a Department of Acute Care, University Medical Centre Groningen, University of Groningen, The Netherlands; b Department of Emergency Medicine, Isala Medical Centre Zwolle, The Netherlands; c Department of Anaesthesiology, University Medical Centre Groningen, University of Groningen, The Netherlands; d Lifeliner 4, Groningen Airport Eelde, University Medical Centre Groningen, The Netherlands; e UMCG Ambulancezorg, Tynaarlo, Drenthe, The Netherlands; f Ambulancezorg Groningen, Groningen, The Netherlands; g Kijlstra Ambulancezorg, Drachten, Friesland, The Netherlands; h Department of Internal Medicine, University Medical Centre Groningen, University of Groningen, The Netherlands; i London’s Air Ambulance Charity, London, United Kingdom

**Keywords:** out-of-hospital cardiac arrest (OHCA), Post-resuscitation care, emergency medical services (EMS)

## Abstract

**Background:**

Early restoration of normal physiology when return of spontaneous circulation (ROSC) is obtained after an out-of-hospital cardiac arrest (OHCA) reduces the risk of developing post-cardiac arrest syndrome (PCAS). This study aims to investigate if (and to which extent) this can be achieved within the scope of practice of standard emergency medical services (EMS) crews.

**Methods:**

A prospective mixed-methods quantitative and qualitative cohort study was performed including adult patients with a non-traumatic OHCA presented to a university hospital emergency department (ED) in the Netherlands after pre-hospital ROSC was obtained. Primary endpoint was the percentage of patients with deranged physiology post-ROSC in whom EMS crews were able to reach recommended treatment targets.

**Results:**

During a 32-month period, 160 patients presenting with ROSC after OHCA were included. Median (IQR) pre-hospital treatment duration was 40 (34–51) minutes. When deranged physiology was present (n = 133), it could be restored by EMS crews in 29% of the patients. Although average etCO_2_ and SpO_2_ improved gradually over time during pre-hospital treatment, recommended treatment targets could not be achieved in respectively 55% (30/55) and 43% (20/46) of the patients. Similarly, airway problems (24/46, 52%), hypotension (20/23, 87%) and post-anoxic agitation (16/43, 37%) could often not be resolved by EMS crews. The ability to restore normal physiology by EMS could not be predicted based on patient characteristics or in-arrest variables.

**Conclusion:**

Deranged physiology after an OHCA is commonly encountered, and often difficult to treat within the scope of practice of regular EMS crews. Involvement of advanced critical care teams with a wider scope of practice at an early stage may contribute to a better outcome for these patients.

## Highlights

• After successful resuscitation following out-of-hospital cardiac arrest, derangements of physiological parameters are common in the immediate pre-hospital phase.
• Within their current scope of practice, regular emergency medical service providers are unable to restore physiological parameters within normal ranges in the majority of cases.
• Higher incidences of physiological derangements were observed in patients resuscitated for extended periods, those with PEA as their initial arrest rhythm, low post-arrest consciousness levels and higher doses of administered adrenalin.
• The threshold to request assistance from advanced pre-hospital care teams should be low, especially when patients experience airway failure, hypotension and/or post-anoxic agitation.

## Graphical abstract

**Figure:**
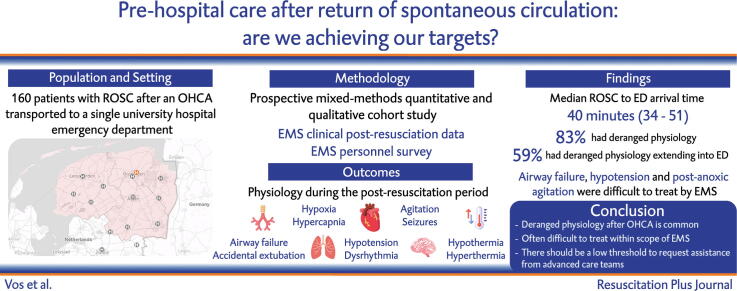

## Introduction

Out-of-hospital cardiac arrest (OHCA) is a condition associated with a high mortality, and chances of neurologically intact survival are directly related to the duration of the arrest.^1^ Therefore, prompt recognition of the cardiac arrest, followed by early (bystander) cardiopulmonary resuscitation (CPR) and early defibrillation (using a public automatic external defibrillator (AED) are a prerequisite for neurologically intact survival.^2^

Once a return of spontaneous circulation (ROSC) is obtained, post-resuscitation care should be started immediately to minimise the effects of whole body ischaemia and reperfusion injury that occur during and after the arrest, which are responsible for the development of a post-cardiac arrest syndrome (PCAS).^3^ PCAS is a clinical state comprising post-anoxic brain injury, myocardial dysfunction, a systemic inflammatory response, microcirculatory failure, adrenal suppression and/or coagulopathy.^4^ As physiological derangements in the post-arrest phase, such as hypoxia, hypercarbia, hypotension, hyperthermia and seizures, have been shown to contribute to the development and severity of PCAS, it is important to aim for early restoration of normal physiology after ROSC is obtained.^5^

This, however, can be challenging in the pre-hospital setting, as therapeutic options for emergency medical service (EMS) crews to treat deranged physiology are often limited as compared to in-hospital. Currently, it is unclear if post-arrest targets as published by the European Resuscitation Council (ERC) and the American Heart Association (AHA) can practically be met within the scope of practice of EMS crews.^3^, ^6^

Therefore, this study aims to investigate how often post-resuscitation care targets can be met in the pre-hospital setting by EMS crews, and which patient-, provider- and treatment factors are related to successful pre-hospital post-cardiac arrest care.

## Methods

### Study design

A single-centre mixed-methods quantitative- and qualitative prospective cohort study was performed to evaluate EMS post-arrest care of all adult OHCA patients who were presented with ROSC after a non-traumatic OHCA to the emergency department (ED) of a large university hospital in the Netherlands (University Medical Centre Groningen, UMCG) between July 1st 2020 and March 1st 2023. Vital signs for the whole pre-hospital treatment phase were prospectively collected for all patients to identify which patients had a period of deranged physiology and in order to quantify the timing- and magnitude of these derangements. Finally, after handover, EMS crews were asked to fill in a questionnaire regarding the quality of post-arrest care they were able to provide within their scope of practice.

### Study setting

The study was performed in the northern part of the Netherlands. In the entire Netherlands, when an emergency call is made for an OHCA, both trained citizen first responders and two EMS crews are dispatched by default to the location of the arrest. EMS crews in the entire country consist of a specialised pre-hospital care nurse (Advanced Life Support (ALS) qualified) and an EMS driver (Basic Life Support (BLS) qualified). All EMS crews are able to provide full Advanced Cardiopulmonary Life Support (ACLS). Refresher courses are followed yearly. A national EMS protocol is in place where EMS crews adhere to. Intra- and post-arrest care guidance in this protocol is in line with the latest ERC guidelines.^3^ Crews are advised to aim for a peripheral blood oxygen saturation (SpO2) > 94% and to prevent hyperoxia, to aim for end-tidal carbon dioxide (etCO2) values between 3.0 and 5.5 kPa, to perform a 12-lead electrocardiogram (ECG), to correct hypoglycaemia, to decompress the stomach with a nasogastric tube, and to immediately transfer the patient to the most appropriate hospital according to local triage protocols. EMS nurses are able perform endotracheal intubations. As per 2020, placement of a supraglottic airway device is preferred over endotracheal intubation to manage the airway, following COVID-19 recommendations. Additionally, oxygen can be supplemented non-invasively or by basic- or positive pressure mechanical ventilation. Nurses can give fluid boluses in the context of hypotension, but are not able to give vasopressors. Tachyarrhythmias can be terminated by synchronised cardioversion or drugs (adenosine or amiodarone) whilst bradycardias can be treated with external pacing and/or atropine. In case of severe agitation or seizures, sedation with midazolam is also within the scope of EMS nurses. Advanced deep sedation and drug-assisted intubations are not performed by EMS and require HEMS assistance.

A physician-staffed HEMS team can be alerted when predefined dispatch criteria (e.g. drowning, traumatic arrest, paediatric arrest) are met or on request of the EMS crew on-scene. In addition to this, with the start of the nationwide Dutch ON-SCENE trial in 2022^7^, HEMS is immediately dispatched to all patients < 50 years with an OHCA. Within the study area, one HEMS team is available 24/7. They respond by helicopter in both day- and nighttime and switch to a quick response car in adverse weather conditions. If the team is unavailable, HEMS from outside the study area can be dispatched.

The study region is covered by three different emergency medical services. As per local guidelines, most patients with ROSC after OHCA are conveyed to one of three centres with percutaneous coronary intervention (PCI) capabilities (supplementary file 1). The study hospital is one of these three centres.

### Study population

Eligible for inclusion into the study were adult patients (age > 18 years) who suffered a non-traumatic OHCA*, who achieved ROSC prior to ED arrival, and who were not in the hospital’s informed opt-out registry for medical research.

*Traumatic cause of cardiac arrest. Asphyxia due to hanging, drowning and electrocutions were not considered traumatic causes in this study, as regular ALS protocols are followed in these situations.

### Clinical endpoints

#### Primary endpoint

The primary endpoint was defined as the percentage of OHCA patients who had deranged physiology or in whom other conditions predisposing to PCAS were present*, wherein EMS crews were able to reach post-ROSC treatment targets** without the involvement of HEMS and before handover in hospital.

* Other conditions predisposing to PCAS were defined as:

- Accidental extubation or actual- or impending airway failure, defined as the absence- or malfunction of a supraglottic airway device or endotracheal tube (ETT) reflected by either the HEMS team (if attending) or the receiving hospital team’s decision to (re)intubate the patient immediately after attending/arrival in the ED;
- Uncontrolled post-anoxic agitation or uncontrolled seizures despite the administration of benzodiazepines.

** Post-ROSC treatment targets (as recommended by the ERC^3^) were defined as:

- No hypoxia (defined as a SpO2 < 94% on at least two consecutive measurements within five minutes);
- No hypercarbia (defined as an etCO2 > 5.5 kPa on at least two consecutive measurements within five minutes);
- No hypotension (defined as a mean arterial pressure (MAP) < 65 mmHg, or the use of vasopressors by a HEMS team (if attending), or the presence of dysrhythmias (potentially) compromising haemodynamics);
- No hyperthermia (body temperature > 38℃) or hypothermia (body temperature < 35℃).

#### Secondary endpoints

- The frequency distribution of individual components of the primary endpoint;
- Duration of physiological derangements in individual patients, measured from the moment of first occurrence until resolved or until arrival in hospital;
- Patient- and resuscitation factors related to deranged physiology in the post-resuscitation phase;
- Self-rated quality of post-arrest care by EMS providers.

### Data acquisition

Pre-hospital data regarding patient- and resuscitation characteristics were collected from the electronic patient files from EMS (Elektronisch Dossier Ambulancezorg, EDAZ). Vital signs, including respiratory rate, SpO2, heart rate, blood pressure and EtCO2 are automatically uploaded from the EMS Corpuls® patient monitor (Corpuls Nederland BV, Hellevoetsluis, the Netherlands) to the EDAZ. Additionally, a short questionnaire regarding the quality of the pre-hospital care was presented to the EMS nurse upon arrival in hospital and collected by the receiving team (supplementary file 2).

Collected EMS data included: patient characteristics (age, sex, medical history, advanced directives), cardiac arrest characteristics (location of arrest, witnessed arrest, bystander CPR, AED use, AED shocks, initial cardiac rhythm on EMS arrival, ROSC status), pre-hospital interventions (type and dose of administered medication, number of defibrillators, airway management), vital parameters over time (etCO2, SpO2, heart rate, blood pressure, Glasgow coma score (GCS)), the presence of agitation, seizures, vomiting or accidental extubation, Utstein timings (EMS dispatch time, arrival on-scene, leaving scene, hospital arrival), and logistical dispositions (extrication difficulties, transportation mode).

### Sample size

As the primary aim of the study was descriptive, sample size was estimated based on our secondary endpoints. In order to test the overall fit of a model (R^2^) with at least 15 predictor categories to predict deranged physiology and/or complications, we aimed for a sample size of 175 post-resuscitation patients.^8^

### Ethical considerations

The study was determined to be exempt research by the institutional medical ethical review board of the UMCG (METc UMCG, nr2019/613). As only routinely collected pseudonymized data were analysed, deferred consent was not obtained from patients and/or relatives (LTC IGK UMCG, nr201900756). The hospital’s opt-out register for medical research was checked for each participant before data were included in the study. Data sharing agreements were signed between the UMCG and the EMS prior to data transfer and analysis.

### Data analysis

#### Statistical analysis

Continuous variables are expressed as mean (95% Confidence Interval (CI)) when normally distributed and otherwise as median with interquartile range (IQR). Categorical data are presented as absolute numbers and percentages. Differences between patients with- and without deranged post-resuscitation physiology were analysed using Student’s *t*-test or the Mann-Whitney *U* test where appropriate. Differences between categorical variables were analysed by using the Chi-squared test or the Fisher’s exact test (depending on the number of subjects). Univariate correlation analysis using point biserial correlation coefficients were carried out to test the prognostic ability of various patient- and resuscitation characteristics to predict post-resuscitation deranged physiology. A multivariate logistic regression analysis with calculation of odds ratio’s (OR) was carried out to investigate which variables with a univariate correlation coefficient > 0.2 contribute independently to the prediction of deranged post-resuscitation physiology. A two-sided *P*-value < 0.05 was considered statistically significant. All statistical analyses were performed using SPSS 29.0 software (Inc., Chicago, IL, USA).

## Results

### Study population and demographics

**Fig. 1:**
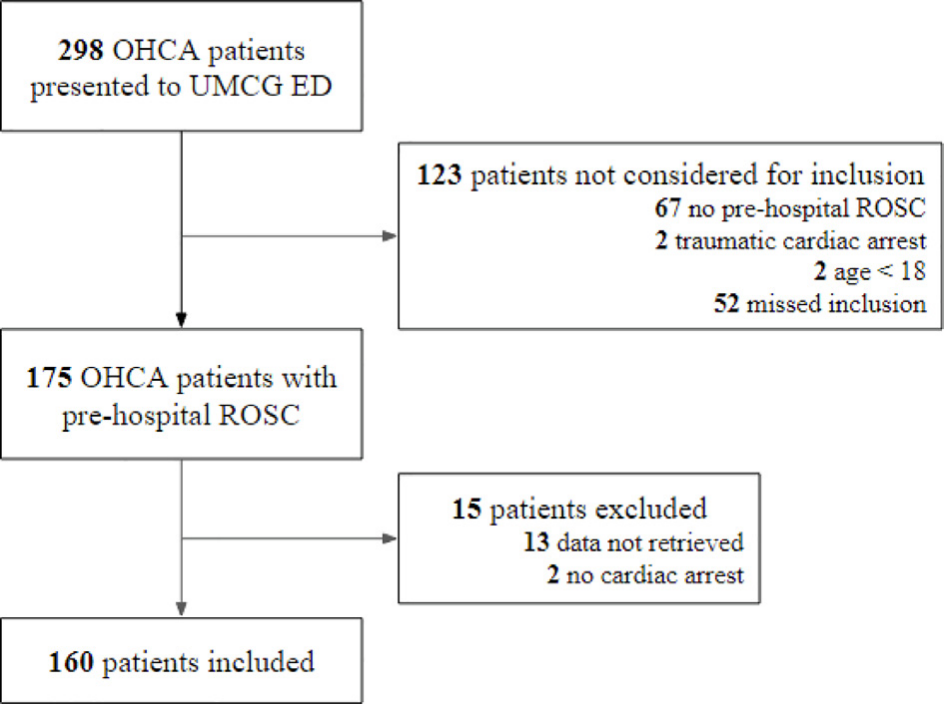
Consort diagram. **Legend figure 1.** OHCA, out-of-hospital cardiac arrest; UMCG, University Medical Centre Groningen; ED, emergency department; ROSC, return of spontaneous circulation.

Between July 2020 and March 2023, 298 patients were brought to the UMCG after an OHCA. 231 of these were presented with ROSC. Two patients were < 18 years, and two had a ROSC after a traumatic cardiac arrest, leaving 227 potentially eligible patients. Of these, 175 (76%) could be included in the present study. Of these, after chart review, the authors discovered that two patients had not been in cardiac arrest, whereas for 13 patients no data regarding the post-cardiac arrest vital signs could be retrieved from the patient records. The study population consisted of the remaining 160 patients (Fig. 1).

Demographics and main characteristics of the study population are represented in Table 1. Most of the patients were male and had a witnessed arrest. The majority received bystander CPR, and in 43% an AED was applied before EMS arrival. ROSC was obtained prior to EMS arrival in 18% of the patients. Most of the patients who were still in arrest when EMS arrived had a shockable rhythm.

**Table 1:** Patient-, arrest- and pre-hospital management characteristics.

**Characteristics**	**Median (IQR) or n (%)**	**Missing n (%)**
Age in years,	64 (55–74)	0 (0)
Sex		
- male	104 (65)	0 (0)
- female	56 (35)	
*Clinical arrest characteristics*		
Witnessed arrest	141 (88)	0 (0)
- witnessed by EMS	16 (10)	
Bystander BLS	138 (86)	0 (0)
Public AED applied	69 (43)	1 (1)
- shock delivered	55 (34)	
EMS response time in minutes	9 (6–11)	7 (4)
Initial EMS rhythm		
- pulseless VT	2 (1)	0 (0)
- VF	71 (44)	
- PEA	37 (23)	
- asystole	21 (13)	
- other*	29 (18)	
*Resuscitation timings*		
Total arrest time, median (IQR), minutes	16 (10–24)	20 (12)**
BLS time, median (IQR), minutes	7 (2–10)	31 (19)
ALS time, median (IQR), minutes	8 (2–14)	12 (7)
*Timing of ROSC*		
- prior to EMS arrival	29 (18)	0 (0)
- before transport to hospital	160 (1 0 0)	
- at handover in hospital	156 (98)	
*Post-arrest neurology*		
GCS (highest recorded)		17 (11)
- 3	87 (54)	
- 4 ≥ 8	24 (15)	
- 9 ≥ 12	12 (8)	
- > 12	20 (13)	
Spontaneous respiration present	113 (71)	9 (6)
Positive pupillary response	105 (66)	12 (7)
*Post-arrest timings*		
EMS on-scene time (minutes)	33 (26–41)	9 (6)
- transport delay due to extrication difficulties	17 (11)	
EMS transport time (minutes)	18 (12–24)	9 (6)
- transport delay due to rendez-vous HEMS	5 (3)	
Pre-hospital post-ROSC period (minutes)	40 (34–51)	7 (4)

**Legend table 1**. Represented are median (IQR) or n (%). * sinus rhythm, atrial fibrillation or supraventricular tachycardia. ** all missing cases were due to non-witnessed arrest. IQR, interquartile range; EMS, emergency medical service; BLS, basic life support; AED, automatic external defibrillator; VT, ventricular tachycardia; VF, ventricular fibrillation; PEA, pulseless electrical activity; ROSC, return of spontaneous circulation; ALS, advanced life support; GCS, Glasgow coma score.

### Primary and secondary outcomes

#### Percentage of OHCA patients meeting physiological treatment targets

Only 27 of the 160 post-arrest patients (17%) met all post-ROSC treatment targets. These patients all had a witnessed arrest with immediate bystander CPR and a shorter than average time to ROSC (*p* < 0.01) with a median of 13 min (IQR 4–18) compared to 17 min (IQR 11–24). Ninety-two percent (46/50) of the patients who were resuscitated for over 20 min and all patients (16/16) who had been in arrest for 30 min or more did not meet one or more of the post-ROSC treatment targets. When deranged physiology or one or more other PCAS predisposing conditions were present (n = 133), these could not be resolved by EMS crews in 71% (n = 94) of the cases without the help from HEMS before the care was handed over in hospital.

#### Frequency distribution and duration of PCAS predisposing conditions

**Fig. 2:**
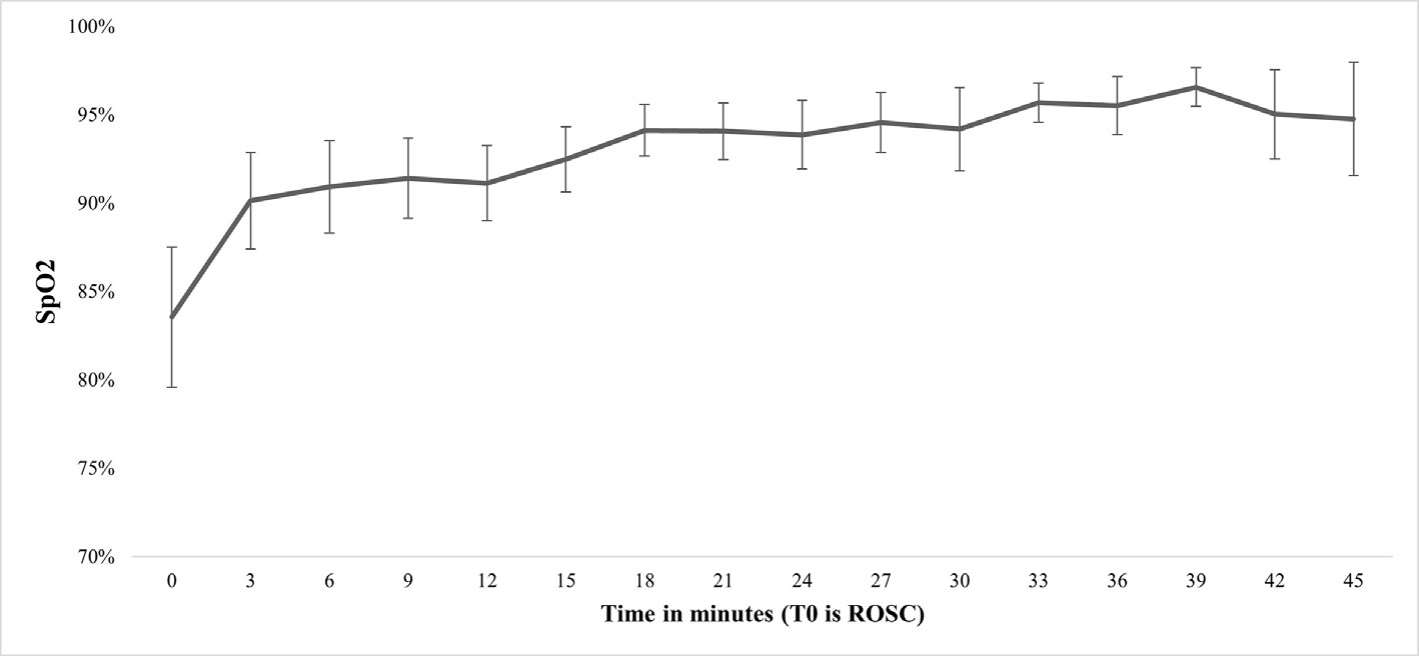
Change in SpO2 over time after ROSC. **Legend figure 2.** Mean (95% CI) peripheral oxygen saturation (SpO2) after return of spontaneous circulation (ROSC).

**Fig. 3:**
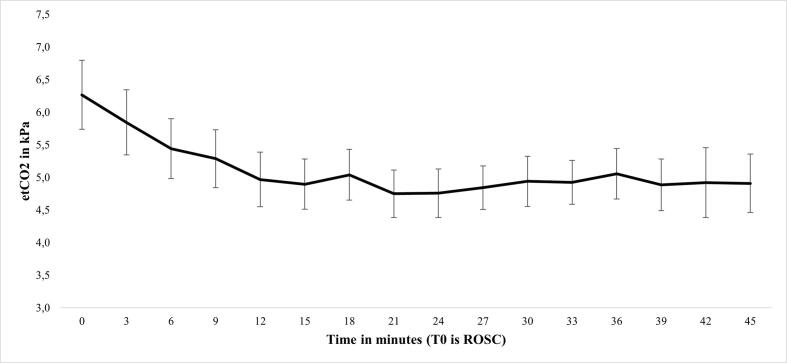
Change in etCO2 over time after ROSC. **Legend figure 3:** Mean (95% CI) end-tidal carbon dioxide (etCO2) prior to- and after return of spontaneous circulation (ROSC).

Actual- or impending airway failure, hypoxia, hypercapnia, re-arrest and agitation in the post-resuscitation phase, were amongst the most frequent PCAS predisposing conditions encountered, each being present in about a third of patients (Table 2.). Most airway issues ((impeding) airway failure or accidental extubation) could not be solved by EMS crews in the pre-hospital setting without the assistance of HEMS. Hypoxia and hypercarbia were frequently encountered, with the highest average etCO2 values and lowest SpO2 values being registered immediately after ROSC. Although both SpO2 and etCO2 demonstrated a tendency to improve over time in the post-resuscitation phase (Fig. 2, Fig. 3), normalisation was difficult to achieve in many patients, and half of the patients had hypoxia and/or hypercarbia on arrival in hospital. Hypotension was observed in 23 (14%) patients and could only be treated successfully in 13% of the patients. Re-arrests occurred in 34 patients (21%) and were treated successfully in 30/34 cases (88%) before hospital arrival, most often within two minutes of the re-arrest. EMS were more successful in terminating pre-hospital tachyarrhythmias than bradyarrhythmias. Uncontrolled agitation was a frequently encountered problem and a minority of patients had seizures. Both could not be resolved by EMS crews in over 50% of cases. Patients meeting Utstein criteria (witnessed arrest, immediate bystander CPR and shockable initial rhythm) had significantly lower rates of hypercapnia but significantly higher rates of agitation (both *p* < 0.01) compared to patients not meeting these criteria (supplementary file 3).

**Table 2:** Frequency distribution of conditions associated with PCAS in the pre-hospital setting, stratified by success of treatment.

	**Condition**	**Incidence** *n (%)*	**Duration in minutes** *median (IQR)*	**Resolved** *n (% of incidence)*	**Missing** *n* (%)
*Airway*	Actual or impending airway failure	46 (29)	30 (20–35)	22 (48)	0 (0)
	accidental extubation	4 (3)	16 (2–32)	0(0)	
*Breathing*	Hypoxia	46 (29)	18 (9–30)	26 (57)	56 (35)
	Hypercapnia	55 (34)	21 (9–36)	25 (45)	50 (31)
*Circulation*	Re-arrest	34 (21)	2 (2–8)	30 (88)	0 (0)
	Hypotension	23 (14)	15 (9–20)	3 (13)	24 (15)
	Bradyarrhythmia	26 (16)	10 (6–15)	15 (58)	14 (9)
	Tachyarrhythmia*	10 (6)	**	10 (1 0 0)	
*Disability*	Agitation	43 (27)	13 (10–26)	27 (63)	0 (0)
	Seizure	5 (3)	8 (6–10)	2 (40)	0 (0)
*Exposure*	Hyperthermia	1 (1)	**	1 (1 0 0)	93 (68)
	Hypothermia	19 (12)	**	6 (32)	

**Legend table 2.** percentages in the frequency column are proportions of total cohort (*n* = 160). A PCAS predisposing condition was regarded as successfully managed when EMS crews were able to normalise physiology or solve the condition without HEMS involvement and prior to hospital arrival. * Most often recurrent VT. ** Durations not reliably recorded. PCAS, post-cardiac arrest syndrome; No., number; IQR, interquartile range; EMS, emergency medical service; HEMS, helicopter emergency medical service.

#### Role of HEMS

HEMS attended 48 of the 160 patients (30%). The team was most frequently dispatched on request of the on-scene EMS crew (33/48, 69%). Median time from dispatch to arrival on-scene was 13 min (IQR 10–15). Interventions provided by HEMS were definitive airway management in 40 cases (83%), advanced sedation (*n* = 28, 58%), ultrasound diagnostics (*n* = 18, 38%) and vasopressor therapy (*n* = 14, 29%). Patients who were attended by HEMS initially had significantly higher rates of PCAS predisposing conditions (96% versus 78%, *p* = 0.01), reflecting that this was a sicker cohort of patients. Patients attended by HEMS also had significantly lower rates of spontaneous respiration (15% versus 58%, *p* < 0.01) after ROSC was achieved. Post-ROSC treatment targets were met in 48% of patients attended by HEMS, which was similar to patients not attended by HEMS (49%, *p* = 0.89). HEMS interventions resulted in significantly higher rates of adequate management in the airway (100% versus 29%, *p* < 0.01) and disability domains (89% versus 47%, *p* = 0.02). No significant differences were observed in the other domains, although SpO2 and etCO2 normalised faster in the HEMS group. HEMS attendance resulted in significantly longer (*p* < 0.01) on-scene times with a median of 42 min (IQR 32–52), compared to 30 min in EMS-only group (IQR 24–36).

#### Patient- and resuscitation factors related to an inability to optimise physiology

Patients with a longer time spent in arrest, PEA as initial rhythm, a lower GCS after ROSC and higher total dose of administered adrenalin were all associated with a higher incidence of PCAS predisposing conditions in univariate correlation analysis (supplementary file 4). In multivariate analysis however, no independent variables could be identified predicting the (in)ability to restore homeostasis before arrival in hospital.

#### Experience of EMS providers

EMS providers were generally content with the quality of post-resuscitation care they had been able to deliver (average self-reported score of 8.3/10). When asked about skills outside their scope of practice that could have contributed to a better outcome for the patient treated, EMS providers most frequently mentioned airway skills for ETT placement, the ability of drug-assisted intubation and advanced sedation options. A more advanced mechanical ventilator (to facilitate assisted ventilation and in order to be able to adjust fraction of inspired oxygen (FiO2)) was mentioned as a piece of equipment that could have contributed to a better outcome for patients.

As a general remark, several EMS crews acknowledged that pre-hospital post-resuscitation care is generally difficult, with many tasks to be performed in a short period of time.

## Discussion

This study demonstrates that deranged physiology post-resuscitation after an OHCA is commonly encountered in the pre-hospital environment, and often difficult to treat within the scope of practice of regular EMS crews. Especially airway compromise (resulting in hypoxia), hypotension and post-anoxic agitation are often sub-optimally managed for extended periods of time, which may contribute to the development of PCAS.

Deranged physiology was present in 83% of all patients with an OHCA in whom ROSC was obtained. The exemptions were patients who had a witnessed arrest with a shockable first rhythm: these patients tended to physiologically do well after their arrest. A longer low-flow time duration was associated with a higher risk of deranged physiology, which is physiologically plausible and in line with previous literature.^9^, ^10^ Hypoxia, hypercarbia and hypotension post-ROSC were all frequently encountered, with incidences of hypoxia, hypercarbia and hypotension that were similar to previously published studies.^11^, ^12^, ^13^, ^14^

Importantly, deranged physiology was not only prevalent, but it was also hard to resolve in the pre-hospital setting by EMS crews. When airway problems were encountered (malfunctioning of oral- or supraglottic airway device or vomiting), assistance from an advanced critical care team (HEMS) was often required, and a significant proportion of those not attended by HEMS were immediately intubated upon ED arrival. Similarly, hypoxia and hypercarbia were difficult to manage: although SpO2 and etCO2 values improved gradually over a period of twenty to thirty minute, in about half of the patients with hypoxia or hypercarbia after ROSC, EMS crews were not able to reach the recommended post-ROSC treatment targets.^3^ As even short periods of hypoxia may affect neurological outcome,^15^, ^16^ more aggressive treatment (for example by drug-assisted intubation and positive pressure ventilation) would have been desirable in these patient. However, this was beyond the scope of practice of regular EMS crews. It should be noted though that even when drug assisted intubation would have been available to the EMS crews, in some patients normalisation of SpO2 would likely not have been reached due to underlying pathology. As the relation between hypercarbia and outcome is less well established (relatively short exposure to mild hypercapnia has been reported not to affect neurological intact survival),^15^, ^17^ the consequences of isolated hypercarbia in the absence of hypoxia should not always automatically prompt aggressive prehospital treatment.

Almost 15% of patients had hypotension after ROSC was obtained. As a strong association exists between (both duration and severity of) hypotension and mortality after OHCA, prompt recognition and management are warranted.^18^ However, in only 14% of the patients with post-ROSC hypotension, EMS crews were able to reach a MAP > 65 mmHg en route to hospital. This can most likely be attributed to the fact that vasopressor therapy is outside the scope of practice of standard EMS crews, and fluid boluses are often insufficient and/or contraindicated.

Post-anoxic agitation could not be managed appropriately in over 60% of occurrences, despite that EMS crews (according to the national protocol) have the option to sedate patients with midazolam. Interestingly, post-anoxic agitation was more frequently observed in the Utstein subcohort and in patients with higher GCS after ROSC. Multiple EMS providers mentioned the lack of further, advanced sedation options in the questionnaire as a contributing factor for not being able to prevent post-anoxic agitation. Adequate management of both agitation and seizures however is highly warranted as both are associated with worse neurological outcomes.^19^

Finally, re-arrests occurred in one-fifth of the patients, usually early in the course of the post-resuscitation phase, which is in line with previous reports.^20^ To prevent re-arrests, restoration of homeostasis before commencing transport to hospital is very important.^21^, ^22^ Transporting the patient to hospital without addressing hypoxia, hypercarbia, hypotension, or post-anoxic agitation places patients at higher risk of re-arresting and may affect neurological intact survival.

Our findings call for the provision of more advanced treatment options in the pre-hospital phase of post-ROSC care. Involvement of advanced critical care teams in the care for patients with ROSC after an OHCA is a way to offer these treatments. These teams provide additional expertise and interventions which may contribute to normalisation or improvement of physiological derangements, reducing the risk of re-arrest and potentially resulting in higher rates of intact neurological survival.^23^ Especially in cases of a compromised airway, post-anoxic agitation and seizures, the HEMS physician is able to provide valuable additional interventions. Furthermore, as was stated by EMS personnel in the questionnaire, the presence of experienced critical care practitioners may provide cognitive relief to EMS crews in the often hectic post-ROSC phase, which may translate in a better treatment for these patients. As it is hard to predict which patients will benefit from the more advanced treatment options advanced critical care teams have to offer from the information available at the time of arrest, there should be a low threshold to dispatch these teams once ROSC is obtained, and post-ROSC treatment targets are not immediately met. Our results show this is feasible from a response time perspective, which was on average 13 min.

Although fear of a delay in transport to definitive care is justified, optimisation of physiology prior to transport should be prioritised in this group of patients, especially when transport distances are long.

### Strengths and limitations

This is the first prospective study systematically evaluating the quality of post-ROSC care EMS crews are able to deliver within their scope of practice. Strengths of this study are its combined quantitative and qualitative design, and the limited number of missing data (despite the well-known challenges of data collection in cardiac arrest research). This study also has some limitations. First, patients in whom ROSC was achieved, but who subsequently lost output again on-scene, were not transported to hospital and hence not included, generating a potential for selection bias. Second, generalizability of our findings may be hampered by the fact that the scope of practice (and with that potential treatment options) may vary between emergency medical services. Thirdly, data collection was reliant on routinely measured physiological parameters, often with automated time intervals. Discrepancies in intervals were observed, which resulted in (a limited number of) missing data points. Furthermore, some measurements were annotated by the crews to be unreliable (e.g. due to cold extremities in the case of peripheral SpO2 or technical malfunctioning) and were therefore not included. Finally, the study took place in the Netherlands, where bystander CPR-, AED application- and ROSC rates are relatively high compared to many other countries.^24^ This has to be taken into account when the results are interpreted.

## Conclusion

Deranged physiology after an OHCA is commonly encountered, and often difficult to treat within the scope of practice of regular EMS crews. Involvement of advanced critical care teams with a wider scope of practice at an early stage may contribute to a better outcome for these patients.

## CRediT authorship contribution statement

**I.A. Vos:** Data curation, Formal analysis, Investigation, Validation, Visualization, Writing – original draft. **F.G. Lucassen:** Conceptualization, Data curation, Investigation, Methodology, Project administration, Software, Writing – review & editing. **B.W.J. Bens:** Conceptualization, Methodology, Writing – review & editing. **B. Dercksen:** Resources, Writing – review & editing. **R. Postma:** Resources, Writing – review & editing. **E.M.F. Jorna:** Resources, Writing – review & editing. **J.C. ter Maaten:** Supervision, Writing – review & editing. **M.M.R.F. Struys:** Supervision, Writing – review & editing. **E. ter Avest:** Conceptualization, Methodology, Project administration, Resources, Supervision, Writing – original draft.

## Declaration of competing interest

The authors declare that they have no known competing financial interests or personal relationships that could have appeared to influence the work reported in this paper.

## Figures and Tables

**Supplementary Fig. 1:**
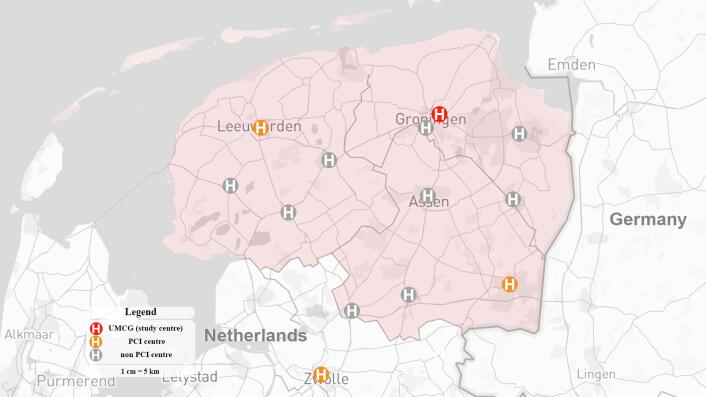
study area. **Legend supplementary file 1**: graphic visualisation of the study area in the northern part of the Netherlands. The study centre is depicted in red. Other PCI centres are depicted in blue, whilst non-cardiac centres are depicted in grey.

## Appendix A Supplementary data

Supplementary File 1 questionnaire for EMS personnel.

Supplementary File 2 Utstein cohort subgroup analysis.

Supplementary File 3 univariate correlation analysis for unresolved predisposing PCAS conditions.
