# Supplementary material for: Pre-hospital care after return of spontaneous circulation: Are we achieving our targets?
Source: Resusc Plus. 2024 Jun 21;19:100691. doi: 10.1016/j.resplu.2024.100691 (PMC11246053; doi:10.1016/j.resplu.2024.100691)
Supplement: Supplementary File 1 — questionnaire for EMS personnel. [file mmc1.docx]

**Supplementary file 2**: questionnaire for EMS personnel

[Translated from Dutch to English]

**Questionnaire for EMS personnel after ED handover**

1. What is the most likely arrest aetiology?

………………………………………..………………………………………..

………………………………………..………………………………………..

1. Are there any aspects of post-resuscitation care that you feel you could not sufficiently take care of? If yes, which ones?

………………………………………..………………………………………..

………………………………………..………………………………………..

………………………………………..………………………………………..

………………………………………..………………………………………..

1. Did you lack any skills or equipment needed to provide optimal post-resuscitation care? If yes, which ones?

………………………………………..………………………………………..

………………………………………..………………………………………..

………………………………………..………………………………………..

………………………………………..………………………………………..

1. How would you rate the quality of post-resuscitation care provided to this patient? (scale 1 – 10).

………………………………………..………………………………………..
