# Supplementary material for: Pre-hospital care after return of spontaneous circulation: Are we achieving our targets?
Source: Resusc Plus. 2024 Jun 21;19:100691. doi: 10.1016/j.resplu.2024.100691 (PMC11246053; doi:10.1016/j.resplu.2024.100691)
Supplement: Supplementary File 2 — Utstein cohort subgroup analysis. [file mmc2.docx]

**Supplementary file 3**: Utstein cohort subgroup analysis

|  | **Condition** | | **Incidence Utstein subcohort** (n = 92)  *n (%)* | **Incidence non-Utstein subcohort** (n = 68)  *n (%)* | ***P*-value** |
| --- | --- | --- | --- | --- | --- |
| *Airway* | Actual or impending airway failure | | 29 (32) | 17 (25) | 0.37 |
|  | accidental extubation | | 4 (4) | 0 (0) | 0.08 |
|  | |  |  |  |  |
| *Breathing* | Hypoxia | | 25 (27) | 21 (31) | 0.61 |
|  | Hypercapnia | | 18 (20) | 37 (54) | **< 0.01** |
|  | |  |  |  |  |
| *Circulation* | Re-arrest | | 18 (20) | 16 (24) | 0.54 |
|  | Hypotension | | 10 (11) | 13 (19) | 0.14 |
|  | Bradyarrhythmia | | 13 (14) | 13 (19) | 0.40 |
|  | Tachyarrhythmia | | 7 (8) | 3 (4) | 0.41 |
|  | |  |  |  |  |
| *Disability* | Agitation | | 34 (37) | 9 (13) | **< 0.01** |
|  | Seizure | | 2 (2) | 3 (4) | 0.42 |
|  | |  |  |  |  |
| *Exposure* | Hyperthermia | | 1 (1) | 0 (0) | 0.39 |
|  | Hypothermia | | 9 (10) | 10 (15) | 0.34 |

**Legend supplementary file 3:** differences in occurrence of PCAS predisposing conditions in patients meeting Utstein criteria versus those who did not. Patients with a witnessed arrest, who received immediate bystander CPR and had a shockable initial rhythm were considered in the Utstein subcohort. Percentages in the frequency column are proportions of the total cohort as depicted.
