# Supplementary material for: Pre-hospital care after return of spontaneous circulation: Are we achieving our targets?
Source: Resusc Plus. 2024 Jun 21;19:100691. doi: 10.1016/j.resplu.2024.100691 (PMC11246053; doi:10.1016/j.resplu.2024.100691)
Supplement: Supplementary File 3 — univariate correlation analysis for unresolved predisposing PCAS conditions. [file mmc3.docx]

**Supplementary file 4**: Univariate correlation analysis for unresolved predisposing PCAS conditions

| **Variable** | **Correlation coefficient (r)** |
| --- | --- |
| Age | -0,059 |
| Gender | 0,055 |
| Hx Diabetes | -0,056 |
| Hx Obesity | -0,038 |
| Hx Hypertension | -0,126 |
| Hx Ischemic heart disease | -0,089 |
| Total arrest time | **0,274** |
| EMS response time | 0,048 |
| Arrest in public domain | -0,146 |
| Witnessed arrest | 0,021 |
| Bystander CPR | -0,062 |
| Bystander AED | 0,018 |
| Initial etCO2 | -0,039 |
| Initial rhythm: ROSC | -0,152 |
| Initial rhythm: shockable | -0,084 |
| Initial rhythm: PEA | **0,200** |
| Initial rhythm: asystole | 0,048 |
| GCS, maximum | **-0,273** |
| Spontaneous respirations | -0,191 |
| Pupil reactivity | -0,104 |
| EMS adrenalin administration | **0,290** |
| EMS invasive airway | -0,139 |
| HEMS involvement | **0,276** |

**Legend Supplementary file 4:** Hx, medical history; EMS, emergency medical service; CPR, cardiopulmonary circulation; AED, automatic external defibrillator; etCO2, end-tidal carbon dioxide; ROSC, return of spontaneous circulation; PEA, pulseless electrical activity; GCS, Glasgow coma score; HEMS, helicopter emergency medical service.
